# Addressing Vaccine Hesitancy to Reduce Racial and Ethnic Disparities in COVID-19 Vaccination Uptake Across the UK and US

**DOI:** 10.3389/fpubh.2021.789753

**Published:** 2021-12-07

**Authors:** Harriett Fuller, Keerthi Dubbala, Dorotheah Obiri, Meryll Mallare, Shailesh Advani, Sophie De Souza, Karlene Stoby, Michelle King-Okoye

**Affiliations:** ^1^The Ethnicity and Covid-19 Research Consortium, International Research Group, Edinburgh, United Kingdom; ^2^Faculty of Environment, School of Food Science and Nutrition, University of Leeds, Leeds, United Kingdom; ^3^Department of Immunology, College of Health Sciences, Noguchi Memorial Institute for Medical Research, University of Ghana, Accra, Ghana; ^4^Nursing Studies, School of Health in Social Science, University of Edinburgh, Edinburgh, United Kingdom; ^5^Affiliate Faculty, Terasaki Institute of Biomedical Innovation, Los Angeles, CA, United States; ^6^School of Population Health and Environmental Sciences, King's College University, London, United Kingdom

**Keywords:** COVID-19, vaccination, ethnicity, vaccine hesitancy, ethnic minorities, healthcare barriers, vaccine uptake, healthcare disparities

## Introduction

Momentous advancements have been achieved in COVID-19 vaccination campaigns within the US and UK; however, COVID-19 continues to have a disproportionate impact on ethnic minority groups (1). COVID-19 has exacerbated existing disparities in healthcare access and outcomes and has a profound effect on the socioeconomic status (i.e., the social class, education, and income status) of minority communities. Despite the amplified burden of COVID-19, vaccine hesitancy remains an issue within ethnic minority groups. The most recent report from the UK's Office of National Statistics (ONS) highlighted that vaccine hesitancy was highest among Black British adults at 21% and has not significantly improved since the start of 2021 (2). Similarly, multiple surveys have called attention to the low uptake of COVID-19 vaccines across the Black population within the US (3). This commentary highlights factors that have contributed to the lower vaccination uptake within ethnic minority groups and provides solutions to address these persisting disparities.

## Drivers of Vaccine Hesitancy Within Minority Communities

### Mistrust Among Ethnic Minority Communities

A considerable barrier contributing to low vaccine uptake within minority groups is a lack of trust towards governments, pharmaceutical companies, and the process of vaccine development. Ethnic minorities in the UK and the US continue to face systemic racism and structural inequalities, which have persisted since the American colonial period and the British Empire (4). Mistrust towards the healthcare system is further driven by negative healthcare experiences and historical medical atrocities, such as the 1932 Tuskegee Syphilis Study in the US where Black men with syphilis were misled into thinking they would receive treatment as part of a government study, but instead were intentionally left untreated to allow for the natural history of untreated syphilis to be observed, even after an effective antibiotic treatment had been discovered. Together, these factors are well established sources of mistrust directed at governments and the healthcare sector that influence vaccine uptake within minority ethnicities (5, 6). Indeed, in a recent study of 101 Black Americans living with HIV, 30% of respondents thought that a COVID-19 cure was being withheld from Black people, whilst only 50% believed that they would receive the same standard of COVID-19 care as other groups (7). Additionally, an April 2021 study of 4,896 UK adults found that 15% more individuals from ethnic minority groups were concerned about the long-term side effects of COVID-19 vaccinations and 8% more were concerned about the ingredients put into the vaccine compared with White participants (8).

### Poor Public Health Messaging

Despite documented vaccine hesitancy among minority communities, recent messaging from the UK government has emphasised an individual's responsibility to be vaccinated, rather than addressing the sources of this hesitancy and mistrust as well as shortcomings in policy decisions, which have contributed to a rise in COVID-19 cases. This fuels the narrative that vaccine hesitancy is a result of ignorance, rather than a result of other complex factors including institutionalised racism and lack of confidence in public health and pharmaceutical companies. Public health messaging leads to outcomes through evoking either negative or positive feelings. It is critical to promote accurate information about the COVID-19 vaccinations in positive ways so as not to incite fear and worry. Open and favourable attitudes are more likely to be achieved when public health messaging strategies are designed in positive ways (9).

### Inequity and Access to COVID-19 Vaccinations

Ethnic minorities can also face unequal and poor access to COVID-19 vaccinations compared with the general population. For example, bureaucratic processes influencing healthcare access alongside waiting times have been reported to be disproportionate for ethnic minorities. Cultural, and linguistic barriers and difficulty travelling due to less access to public transportation or reduced flexibility in working hours, have also been identified as additional barriers for minority communities (7, 8, 10). Despite this, it has been noted that research into the causes of COVID-19 vaccine hesitancy within these communities has not explored the factors influencing hesitancy in depth and how these known barriers contribute to vaccine hesitancy (11). Data at national and local levels are required to establish the proportion of vaccine hesitancy attributable to healthcare barriers and the indirect effect of social determinants, to determine which barriers are the most influential so effective solutions can be facilitated (4). In addition, it is critical that identified barriers in healthcare access are addressed so all individuals receive the same standard of care, independent of their ethnicity.

### Religious Beliefs and COVID-19 Vaccinations

Religion plays an important role in COVID-19 vaccination uptake, particularly within minority groups that can often hold strong religious beliefs. For example, a recent US study found that minority ethnic groups are more likely to be vaccine hesitant based on their religious affiliation (12). Similarly within the UK, adults aged over 70 years who identified as Muslims and Buddhists were the least vaccinated compared with other religious affiliations by March 2021 (13). Studies have identified that faith-based approaches are important in improving vaccine acceptance among vaccine hesitant communities. Holding strong religious beliefs can also present specific ethical concerns regarding vaccination and can contribute to the spread of misinformation and conspiracy theories (12). For example, some Christian sects hold the belief that COVID-19 vaccinations are associated with the “Mark of the Beast” as seen in the Holy Bible (14). Furthermore, the use of foetal cell lines in the developmental stage of COVID-19 vaccines has been misconstrued and has escalated into misinformation that states aborted foetal cells are contained within all COVID-19 vaccines themselves. This misinformation has further driven hesitancy within some Christian groups, which can also be driven by pro-life beliefs (15). The idea of salvation can also be very important for those of a Christian faith, with some individuals believing that accepting a COVID-19 vaccine is also an acceptance of eternal damnation. Furthermore, within Muslim communities there can be concern over the halal status of vaccines, with 11% of Muslims reporting vaccine hesitancy, more so than any other religious group (2). Although some of this misinformation has been debunked, public health engagements must address the concerns of religious minorities to effectively combat vaccine hesitancy. In addition, religious leaders are pivotal in relaying trusted information for vaccine acceptance, particularly within ethnic minority communities, mostly due to a trusting relationship and respect for religious authority, and this can enable informed decision-making (12).

### The Use of mRNA Vaccines

The novel nature of mRNA vaccines has also generated mistrust and heightened fears towards COVID-19 vaccines, particularly within ethnic minorities that have historically not experienced transparent and ethical medical interventions (6). The lack of familiarity with mRNA and vector-based vaccines has contributed to fears that both approaches may alter an individual's DNA (including their autonomy and personality) and could affect fertility. There has also been worrying amounts of misinformation regarding COVID-19 vaccination and the newer vaccine types, particularly online. Common fears include the belief that vaccination will cause shedding of harmful viral and genetic particles, that the inclusion of modern technology within the mRNA vaccines can cause magnetism, and that tracking devices will be injected during vaccination (16). Information campaigns that are designed to reach ethnic minority communities are essential to improve the public's understanding of and provide reassurance around the safety and efficacy of mRNA and other COVID-19 vaccines.

### Inability to Choose Specific COVID-19 Vaccines

In a survey of nearly 5,000 UK adults conducted in April 2021, 60% expressed a preference for a specific COVID-19 vaccine (8). For minority communities, choice is a key contributor to vaccine hesitancy and there are fears that poorer communities will receive less of a choice in which vaccine they receive compared with wealthier individuals who may receive “better vaccines” (17). Ethnic minorities may have less choice over which vaccine they receive due to factors such as distribution plans, meaning that better served areas may receive more vaccines than disadvantaged communities, or as an indirect result of greater barriers to accessing vaccination (i.e., in terms of difficulty with making/rescheduling appointments without monetary or logistical concerns, inability to travel long distances for a vaccine) (17). Furthermore, with many US states now adopting monetary incentives to encourage vaccination, there is a concern that such incentives are coercive (18), particularly to those of low socioeconomic status and people of colour who have been disproportionately impacted by the pandemic. Although such incentives may improve uptake, they can cause further mistrust within communities who already feel they have limited choice regarding vaccination. Vaccine supply now outweighs vaccine demand across much of the US and is expected to increase over the UK over the coming months, meaning supply should no longer be a barrier in preventing vaccine choice (19, 20). An increase in autonomy and acceptance of an individual's preference towards a certain vaccine, whether this be a result of vaccine nationalism, religious beliefs, suspected side effects or trust in newer technologies is crucial in improving uptake, particularly in communities historically disfranchised by public health (8, 17).

### Recommendations

**Figure 1:**
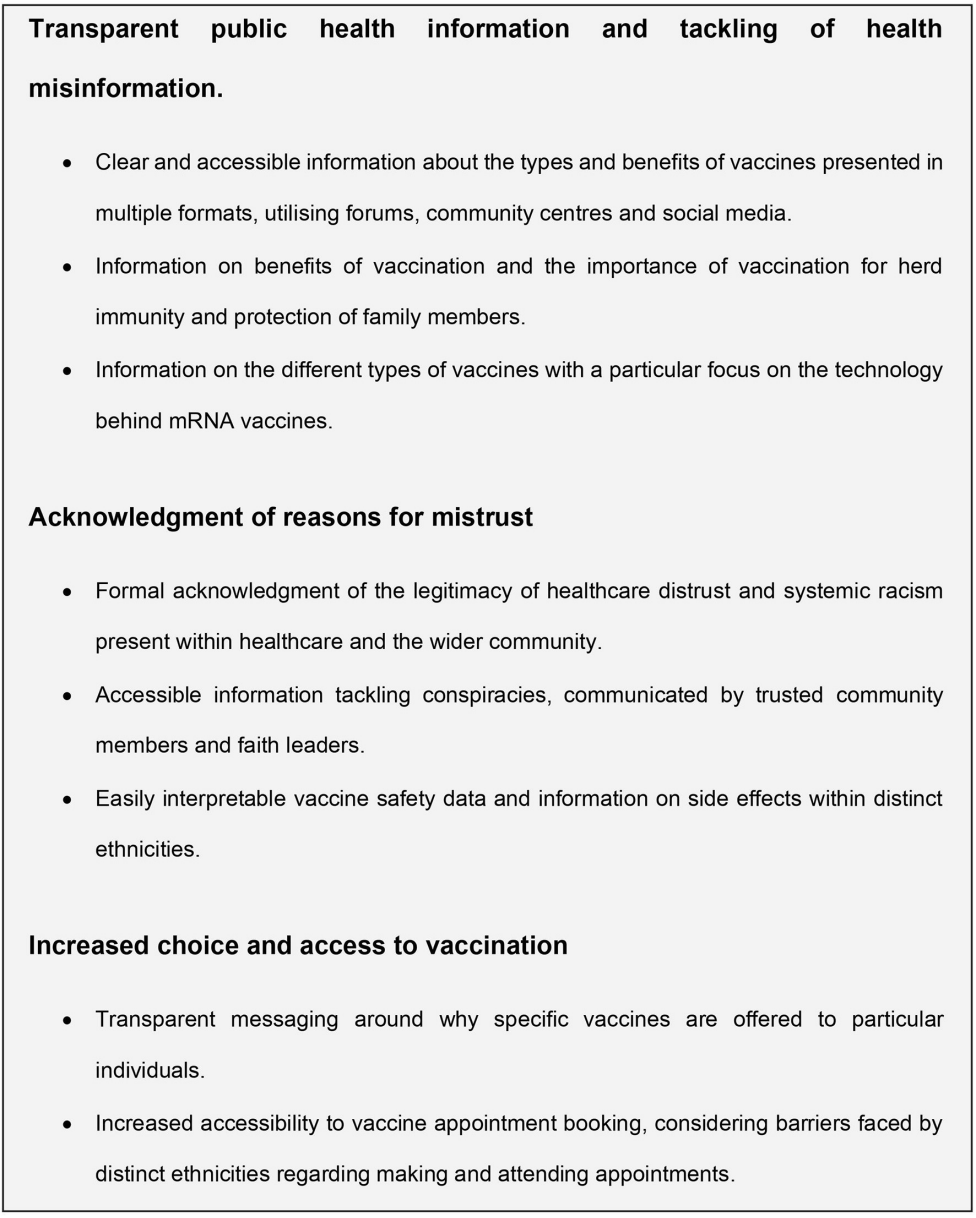
Policy recommendations to improve vaccine uptake in minority communities within the UK and US.

Eradicating racism as a long-term outcome is not easily achievable, but utilising empathy in public health messaging, providing accessible information on vaccine types and working together with religious leaders and faith communities is a steppingstone in the right direction. A summary of key recommendations to improve vaccine uptake among minority ethnic communities within the UK and US is shown in Figure 1.

## Conclusions

In summary, there are multiple, interconnected reasons underlying vaccine hesitancy among minority populations across the UK and the US, including systematic barriers, religious beliefs, vaccine choice and public health messaging. As vaccination is currently our best way to combat the pandemic and protect people from serious illness, it is essential that these reasons are understood and addressed through policy and outreach.

## Author Contributions

MM contributed to the section on 'Mistrust' across ethnic minority communities within the US and UK. KD focused on the section inequity and poor access to COVID vaccinations. SDS contributed on public health messaging and longstanding racism. DO contributed on religious beliefs and COVID-19 vaccinations. SA helped with mRNA COVID-19 vaccinations. KS made edits to the final document. HF contributed to the overall structure, made edits to overall document, and also completed the section on vaccine choice and assisted with formulating solutions to address low uptake and vaccine hesitancy. MK-O contributed on the idea to write this opinion piece, contributed to overall structure and feedback to each member's contribution, and also completed the section on solutions to address low uptake and vaccine hesitancy. All authors contributed to the article and approved the submitted version.

## Conflict of Interest

The authors declare that the research was conducted in the absence of any commercial or financial relationships that could be construed as a potential conflict of interest.

## Publisher's Note

All claims expressed in this article are solely those of the authors and do not necessarily represent those of their affiliated organizations, or those of the publisher, the editors and the reviewers. Any product that may be evaluated in this article, or claim that may be made by its manufacturer, is not guaranteed or endorsed by the publisher.
